# Production of Poly(ε-Caprolactone)/Hydroxyapatite Composite Scaffolds with a Tailored Macro/Micro-Porous Structure, High Mechanical Properties, and Excellent Bioactivity

**DOI:** 10.3390/ma10101123

**Published:** 2017-09-22

**Authors:** Jong-Woo Kim, Kwan-Ha Shin, Young-Hag Koh, Min Jin Hah, Jiyoung Moon, Hyoun-Ee Kim

**Affiliations:** 1 Department of Biomedical Engineering, Korea University, Seoul 136-701, Korea; hiidong98@naver.com (J.-W.K.); kwanha7810@naver.com (K.-H.S.); 2 Department of Public Health Sciences, BK21PLUS Program in Embodiment: Health-Society Interaction, Graduate School, Korea University, Seoul 136-701, Korea; 90minjin@hanmail.net; 3 Institute for BioMaterials, Korea University, Seoul 136-701, Korea; answldud8503@naver.com; 4 Department of Materials Science and Engineering, Seoul National University, Seoul 151-742, Korea; kimhe@snu.ac.kr

**Keywords:** 3D plotting, porous scaffolds, poly(ε-caprolactone), hydroxyapatite, cytocompatibility

## Abstract

We produced poro-us poly(ε-caprolactone) (PCL)/hydroxyapatite (HA) composite scaffolds for bone regeneration, which can have a tailored macro/micro-porous structure with high mechanical properties and excellent in vitro bioactivity using non-solvent-induced phase separation (NIPS)-based 3D plotting. This innovative 3D plotting technique can create highly microporous PCL/HA composite filaments by inducing unique phase separation in PCL/HA solutions through the non-solvent-solvent exchange phenomenon. The PCL/HA composite scaffolds produced with various HA contents (0 wt %, 10 wt %, 15 wt %, and 20 wt %) showed that PCL/HA composite struts with highly microporous structures were well constructed in a controlled periodic pattern. Similar levels of overall porosity (~78 vol %) and pore size (~248 µm) were observed for all the PCL/HA composite scaffolds, which would be highly beneficial to bone tissue regeneration. Mechanical properties, such as ultimate tensile strength and compressive yield strength, increased with an increase in HA content. In addition, incorporating bioactive HA particles into the PCL polymer led to remarkable enhancements in in vitro apatite-forming ability.

## 1. Introduction

There have been great advances in the design and production of porous scaffolds, with mechanical and biological functions tailored to specific bone defects owing to the use of solid free-form fabrication (SFF) techniques [1,2,3,4,5]. More specifically, SFF techniques can selectively consolidate the successive layers of materials or add materials in a layer-by-layer sequence according to predetermined 3-D designs. This unique process can endow porous scaffolds with tightly controlled porous structures (e.g., porosity, pore configuration, pore size, and interconnection between the pores) [2]. Accordingly, the mechanical properties and bone regeneration ability of porous scaffolds would be maximized for bone tissue regeneration [2,3].

As a scaffold material for bone regeneration, poly(ε-caprolactone) (PCL) polymer has been extensively examined owing to its excellent mechanical properties, good cytocompatibility, and reasonable biodegradation rate [6,7]. So far, a variety of SFF techniques have been developed to produce porous PCL scaffolds with controlled porous structures, including selective laser sintering [8,9], 3D plotting [10,11,12,13,14], and fused deposition modelling [15,16], thus demonstrating their great potential for use as bone scaffolds. In addition, the incorporation of bioactive, stiff inorganic materials (e.g., calcium phosphate (CaP) ceramics, and bioactive glasses) into PCL polymer can lead to significant enhancements in mechanical properties, bioactivity, and bone regeneration ability in vivo [8,9,10,11]. Compared to the relatively dense struts, only a few attempts have been made to create microporous struts [17,18], despite their potential to more closely mimic the hierarchical architecture of native bones on the macro- and micro-scales [19,20].

**Figure 1:**
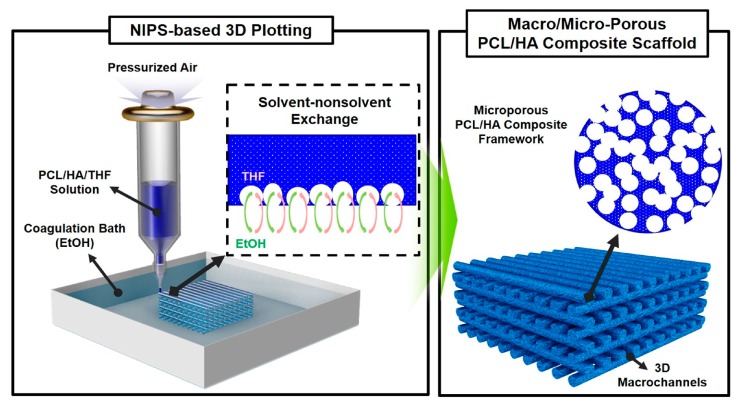
Schematic diagram of the non-solvent induced phase separation (NIPS)-based 3D plotting technique for the production of poly(ε-caprolactone)(PCL)/hydroxyapatite (HA) scaffolds with a controlled macro/micro-porous structure.

In this study, we produced porous PCL/HA composite scaffolds with a tailored macro/micro-porous structure using non-solvent induced phase separation (NIPS)-based 3D plotting, which can provide enhanced mechanical properties, cytocompatibility, and bioactivity compared to the pure PCL scaffold. A schematic diagram of the NIPS-based 3D plotting technique, the basic concept of which was recently developed by our group [18], is illustrated in Figure 1.

A PCL/HA composite solution is prepared by uniformly dispersing HA particles in a PCL/tetrahydrofuran (THF) solution and then extruded through a fine nozzle in an ethanol (EtOH) bath. This deposition process can rapidly solidify PCL/HA filaments with a unique phase separation induced by the exchange of the solvent (THF) and non-solvent (EtOH) [21,22,23,24,25,26], thus enabling the creation of highly microporous PCL/HA composite filaments [16]. These PCL/HA filaments can be deposited in a layer-by-layer sequence according to a predetermined 3D design, and thus 3-D macrochannels separated by highly microporous struts can be constructed. The effect of the HA content on the development of the macro/micro-porous structure, mechanical properties, in vitro cytocompatibility, and apatite-forming ability of porous PCL/HA scaffolds was examined.

## 2. Results and Discussion

### 2.1. Macroporous Structure of Porous PCL/HA Composite Scaffolds

**Figure 2:**
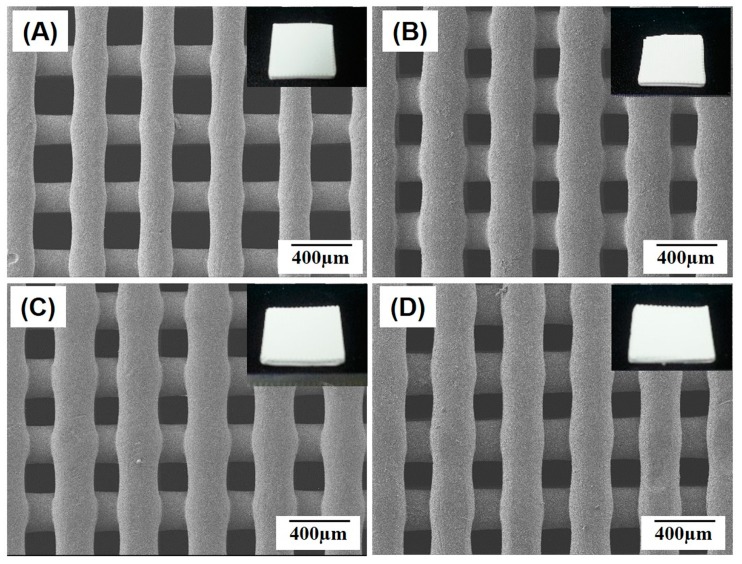
Representative field emission scanning electron microscopy (FE-SEM) images of the macro/micro-porous PCL/HA composite scaffolds produced with various HA contents of (**A**) 0 wt %; (**B**) 10 wt %; (**C**) 15 wt %; and (**D**) 20 wt %, showing the construction of straight PCL/HA struts and macrochannels. Insets in Figure 2A–D show representative optical images of the porous scaffolds.

Porous PCL/HA composite scaffolds with a controlled macro/micro-porous structure were successfully produced using NIPS-based 3D plotting technique, in which three-dimensionally interconnected macrochannels were constructed through the 3-D deposition of highly microporous PCL/HA filaments in a periodic pattern. Regardless of the HA contents, all of the PCL/HA composite scaffolds produced with various HA contents (0 wt %, 10 wt %, 15 wt %, and 20 wt %) demonstrated a predetermined 3D shape without noticeable distortion (insets in Figure 2A–D). In addition, straight PCL/HA composite filaments were well deposited in a controlled periodic pattern for all of the produced scaffolds, as shown in Figure 2A–D. This finding suggests that the incorporation of HA particles into a PCL solution would have negligible influence on the solidification behavior of the PCL/HA composite solutions by means of non-solvent-solvent exchange.

**Figure 3:**
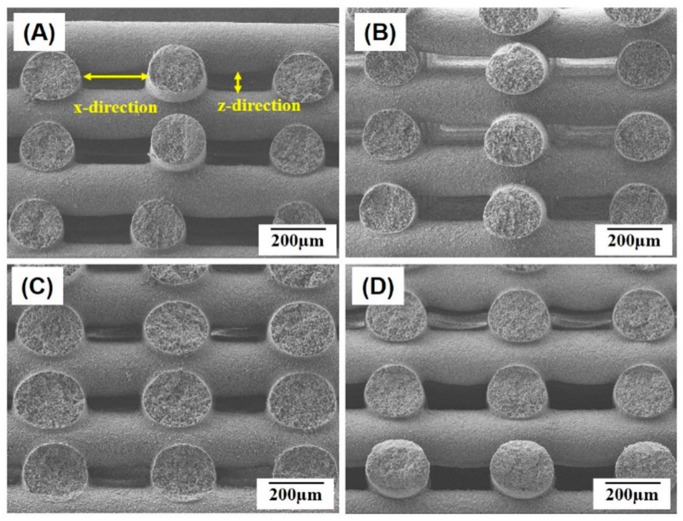
Representative FE-SEM images of the macro/micro-porous PCL/HA composite scaffolds produced with various HA contents of (**A**) 0 wt %; (**B**) 10 wt %; (**C**) 15 wt %; and (**D**) 20 wt %, showing the cross-sectional morphology.

Figure 3A–C illustrate representative cross-sectional images of the porous PCL/HA composite scaffolds produced with various HA contents (0 wt %, 10 wt %, 15 wt %, and 20 wt %). Basically, all of the PCL/HA composite struts comprising scaffolds had a round geometry without severe distortion, which would be attributed to the rapid solidification of the PCL/HA solutions through non-solvent-solvent exchange. However, it should be noted that the PCL/HA filaments would not be completely solidified during deposition onto the previous layers [18]. Thus, the PCL/HA filaments could be slightly merged at the junctions, which would provide strong bonding between the PCL/HA struts.

The dimensions of the PCL/HA composite filaments and macrochannels produced with various HA contents (0 wt %, 10 wt %, 15 wt %, and 20 wt %) were roughly measured based on the SEM images of the scaffolds and are summarized in Table 1. The PCL/HA composite scaffolds had larger diameters than the pure PCL scaffolds, presumably due to lower levels of shrinkage during exchange of the solvent (THF) and non-solvent (EtOH) in the PCL/HA composite solutions. This led to a decrease in the sizes of the macrochannels in both the x- and z-directions (Figure 3A). However, it should be noted that all of the scaffolds had sufficiently large dimensions (in the range of 184 µm × 74 µm–247 µm × 83 µm) that were sufficient for favorable bone tissue regeneration [19,20].

### 2.2. Microporous Structure of PCL/HA Composite Filaments

**Figure 4:**
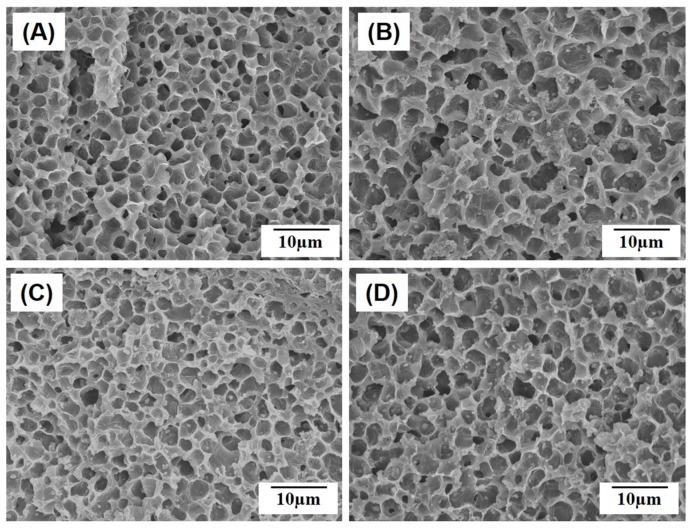
Representative FE-SEM images of the highly microporous PCL/HA composite struts produced with various HA contents of (**A**) 0 wt %; (**B**) 10 wt %; (**C**) 15 wt %; and (**D**) 20 wt %.

One of the most striking features of NIPS-based 3D plotting technique is the ability to create microporous PCL/HA composite filaments by means of exchange of the solvent (THF) and the non-solvent (EtOH) during deposition process [16]. All of the PCL/HA scaffolds produced with various HA contents (0 wt %, 10 wt %, 15 wt %, and 20 wt %) had a highly microporous structure, as shown in Figure 4A–D. A number of micropores were uniformly formed throughout the PCL/HA filaments, while the HA particles were well distributed in the PCL/HA composite struts without noticeable agglomerations. These micropores would be expected to provide favorable paths for mass transport and a large surface area for tissue regeneration in vivo when used as bone scaffolds [19,20]. The final contents of the HA incorporated into the PCL/HA composite scaffolds were 10.1 wt %, 12.1 wt %, and 25.2 wt % for the initial HA contents of 10 wt %, 15 wt %, and 20 wt %, respectively.

All of the scaffolds produced in this study had similar ranges of pore size and overall porosity (Table 1) regardless of the HA contents. This would be attributed to the fact that the fraction and pore size of micropores that can be achieved using non-solvent-solvent exchange should be primarily determined by the polymer concentration [18,21]. Thus, the microporous structure of porous/HA composite scaffolds produced using NIPS-based 3D plotting would be tuned if necessary by adjusting PCL concentration.

### 2.3. Mechanical Properties

**Figure 5:**
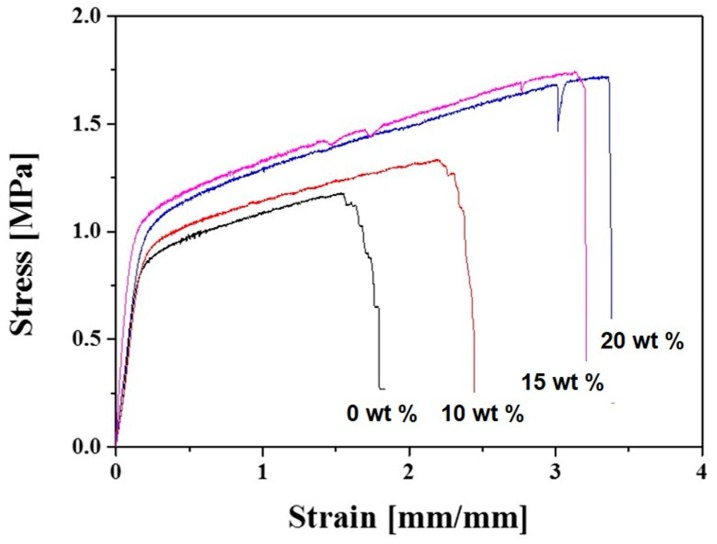
Representative stress versus strain responses of the macro/micro-porous PCL/HA composite scaffolds produced with various HA contents (0 wt %, 10 wt %, 15 wt %, and 20 wt %) under tension.

To evaluate the structural integrity of the macro/micro-porous PCL/HA composite scaffolds as bone scaffolds, their mechanical properties were characterized using tensile strength tests. Figure 5A–D display representative stress versus strain responses of the macro/micro-porous PCL/HA composite scaffolds produced with various HA contents (0 wt %, 10 wt %, 15 wt %, and 20 wt %). Basically, all of the scaffolds demonstrated similar fracture behaviors, namely, an initial elastic response, followed by a considerable plastic deformation before fracture, which is a typical characteristic of highly porous polymers [27].

**Figure 6:**
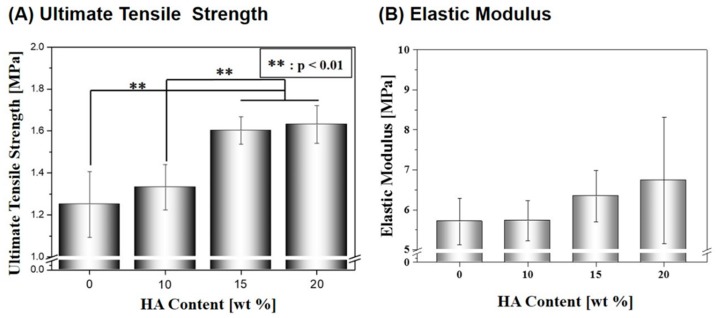
(**A**) Ultimate tensile strength and (**B**) elastic modulus of the macro/micro-porous PCL/HA composite scaffolds (HA content = 0 wt %, 10 wt %, 15 wt %, and 20 wt %).

However, ultimate tensile strength and elastic modulus was increased by the incorporation of the stiff HA particles into the PCL polymer, as shown in Figure 6A,B. As the HA content increased from 0 wt % to 20 wt %, the ultimate tensile strength and elastic modulus increased from 1.25 ± 0.2 MPa to 1.63 ± 0.1 MPa (Figure 6A) and 5.71 ± 0.6 MPa to 6.73 ± 1.6 MPa (Figure 6B), respectively. It should be noted that the composite scaffolds produced with high HA contents of 15 wt % and 20 wt % showed higher ultimate tensile strengths than those produced HA contents of 0 wt % and 10 wt %, which were statistically significant (*p* < 0.01).

**Figure 7:**
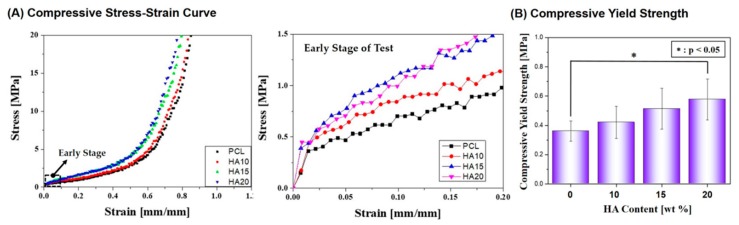
(**A**) Representative stress versus strain responses of the macro/micro-porous PCL/HA composite scaffolds produced with various HA contents (0 wt % (PCL), 10 wt % (HA10), 15 wt % (HA15), and 20 wt % (HA20)) under compression and (**B**) compressive yield strength of the scaffolds. The right graph in Figure 8A displays the stress versus strain responses during the early stage of compressive loading.

In addition, compressive strength tests were conducted for the macro/micro-porous PCL/HA composite scaffolds. Figure 7A shows representative compressive stress versus strain responses of the composite scaffolds produced with various HA contents (0 wt %, 10 wt %, 15 wt %, and 20 wt %). All of the scaffolds showed a typical characteristic of ductile polymers, that is, an initial elastic response was followed by a plateau and rapid increase in stress due to densification [27,28,29]. The compressive yield strength increased significantly from 0.36 ± 0.066 MPa to 0.57 ± 0.136 MPa with an increase in HA content from 0 wt % to 20 wt %, as shown in Figure 7B.

The uniform distribution of stiff HA particles in the PCL polymer is the likely reason for enhancements in mechanical properties, including ultimate tensile strength and compressive yield strengths. More specifically, HA phase can have much higher stiffness than flexible PCL polymer, thus more effectively retaining applied loads, as is often the case with organic/inorganic composites [28,29].

### 2.4. Cytocompatibility

**Figure 8:**
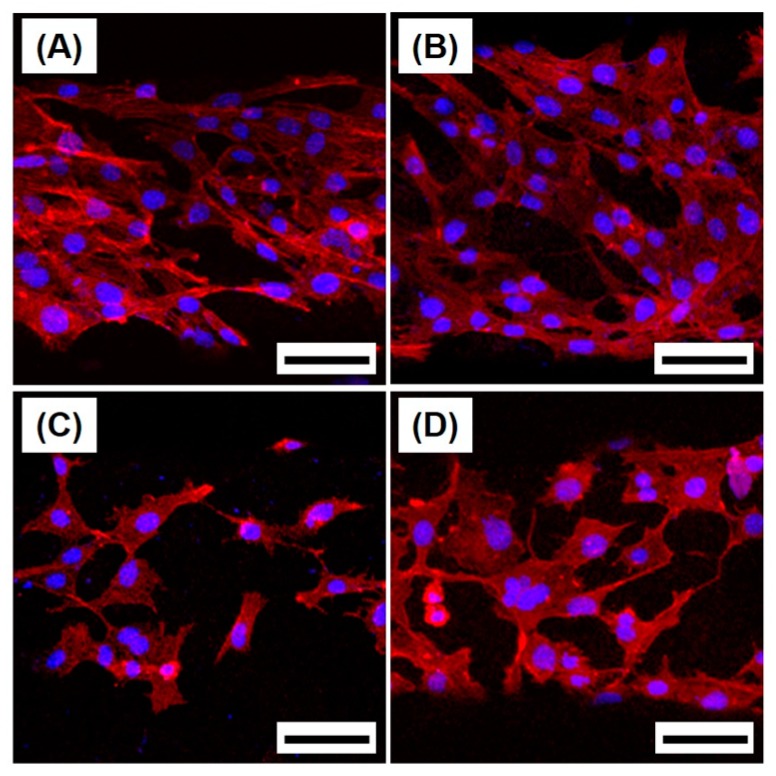
Representative confocal laser scanning microscopy (CLSM) images of the MC3T3-E1 cells on macro/micro-porous PCL/HA composite scaffolds produced with various HA contents of (**A**) 0 wt %; (**B**) 10 wt %; (**C**) 15 wt %; and (**D**) 20 wt % after 24 h of cell culturing (scale = 100 µm).

The cytocompatibility of the macro/micro-porous PCL/HA composite scaffolds was assessed by in vitro cell tests in terms of attachment, proliferation, and differentiation of MC3T3-E1 cells. Figure 8A–D shows representative CLSM images of the MC3T3 cells attached on the PCL/HA scaffolds produced with various HA contents (0 wt %, 10 wt %, 15 wt %, and 20 wt %) after 24 h of culturing, where the red and blue colors represent the actin and nucleus, respectively. Basically, the cells adhered to and spread actively on the surfaces of the scaffolds, suggesting good cytocompatibility.

**Figure 9:**
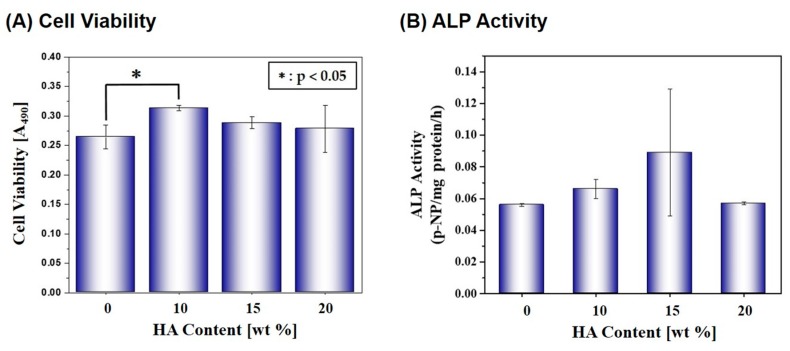
(**A**) Cell viability and (**B**) ALP activity of the MC3T3-E1 cells that were cultured for 5 days and 7 days, respectively, on the macro/micro-porous PCL/HA composite scaffolds (HA content = 0 wt %, 10 wt %, 15 wt %, and 20 wt %).

The effect of the HA content in the PCL/HA composite struts on the cell proliferation and differentiation was examined by MTS assay and ALP activity, respectively, as shown in Figure 9A,B. After 5 days of cell culturing, the scaffold with an HA content of 10 wt % showed the highest level of cell viability, which is statistically significant (*p* < 0.05) when compared to other scaffolds. In addition, after 7 days of cell culture, the composite scaffolds showed slightly higher ALP activities than the pure PCL scaffold; however, no statistically significant differences were observed between the scaffolds.

The ALP activity, which was evaluated using a basal medium instead of an osteogenic culture media, showed a similar trend with a little statistically significant difference, as summarized in Table 2.

These findings suggest that all of the PCL/HA composite scaffolds have good cytocompatibility in vitro; however, the incorporation of the bioactive HA particles would be expected to enhance the biocompatibility in vivo when used as scaffolds [30,31]. In addition, longer periods of time for cell culturing would be required to more clearly demonstrate the utility of bioactive HA phase. It should be also noted that a number of micropores created in the PCL/HA filaments, uniquely obtained in this study, would provide favorable paths for mass transport and large surface areas for cell attachment, proliferation, and differentiation, thus leading to fast bone tissue regeneration in vivo [19,20].

### 2.5. In Vitro Apatite-Forming Ability

**Figure 10:**
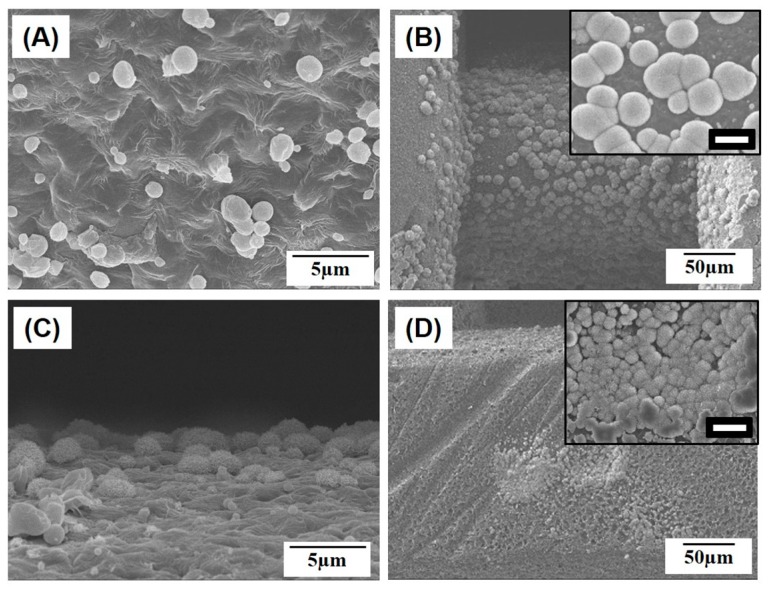

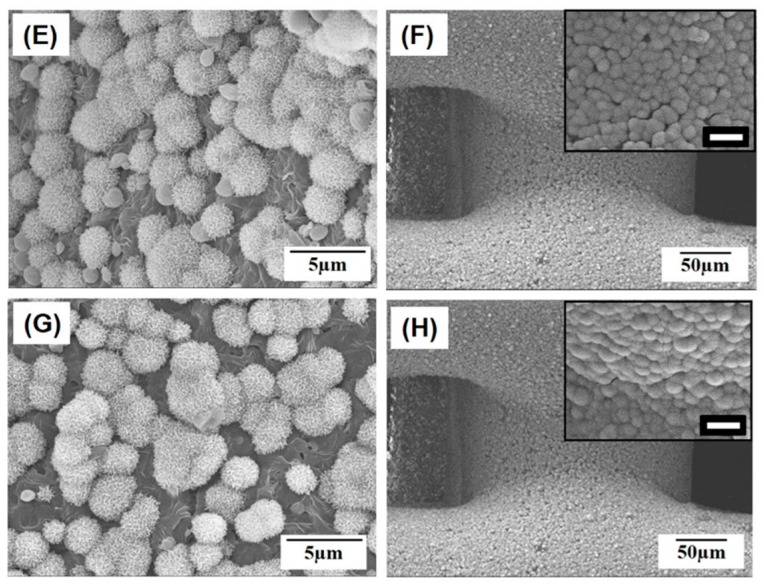
Representative SEM images of the macro/micro-porous PCL/HA composite scaffolds with various HA contents of 0 wt % (**A**,**B**); 10 wt % (**C**,**D**); 15 wt % (**E**,**F**); and 20 wt % (**G**,**H**) after soaking in an simulated body fluid (SBF) solution for 3 days (**A**,**C**,**E**,**G**) and 7 days (**B**,**D**,**F**,**H**). (Scale in insets = 20 µm). Insets show the high-magnification SEM images of the struts.

To evaluate the potential of the macro/micro-porous PCL/HA composite scaffolds for use in bone tissue regeneration, in vitro apatite-forming ability was examined with a simulated body fluid (SBF) test, which is an indicator of the in vivo bioactivity of biomaterials [32,33]. Figure 10A–H presents representative SEM images of the macro/micro-porous PCL/HA composite scaffolds produced with various HA contents (0 wt %, 10 wt %, 15 wt %, and 20 wt %) after soaking in the SBF solution for 3 days and 7 days. The pure PCL scaffold showed negligible apatite crystal precipitation after soaking in the SBF solution for 3 days (Figure 10A). On the other hand, even with the lowest HA content of 10 wt %, apatite crystals started to precipitate on the surface of the PCL/HA composite struts (Figure 10C). This precipitation of apatite crystals became more vigorous as the HA content increased (Figure 10E,G). After the soaking for 7 days, the pure PCL scaffold also showed the precipitation of apatite crystals (Figure 10B); however, the surfaces of the PCL strut were partially covered with apatite crystals (the inset in Figure 10B). The surfaces of the PCL/HA composite struts with high HA contents of 15 wt % and 20 wt % were entirely covered with apatite crystals (the insets in Figure 10F,H).

**Figure 11:**
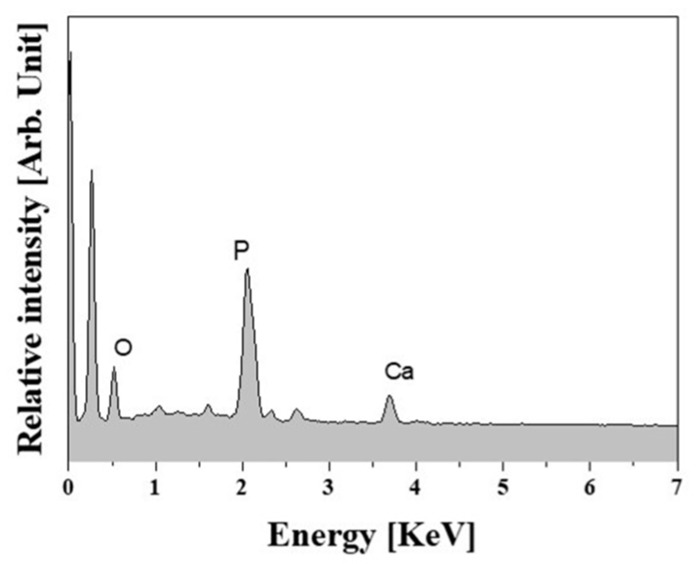
Representative EDS spectrum of the macro/micro-porous PCL/HA composite scaffold produced with a HA content of 20 wt % after soaking in an SBF solution for 3 days.

EDS analyses revealed strong peaks corresponding to Ca, P, and O elements, confirming the precipitation of apatite crystals, as shown in Figure 11. This finding suggests that the bioactivity of the macro/micro-porous PCL/HA composite scaffolds can be enhanced significantly through the introduction of the bioactive HA particles into the PCL polymer.

## 3. Materials and Methods

### 3.1. PCL/HA Composite Solutions Preparation

Unless specified otherwise, all reagents were purchased from Sigma-Aldrich (Sigma Aldrich, St. Louis, MO, USA). PCL solutions at a concentration of 10% *w/v* were prepared by dissolving PCL pellets (Mn = 80,000) in tetrahydrofuran (THF) at 40 °C using magnetic stirring for 24 h. Subsequently, the predetermined amounts of HA powders (OssGen Co., Daegu, Korea) with a mean particle size of ~0.5 µm were added to the PCL/THF solutions to prepare PCL/HA composite solutions with various HA contents (10 wt %, 15 wt %, and 20 wt % in relation to the PCL polymer), followed by magnetic stirring for 24 h at 40 °C.

### 3.2. PCL/HA Composite Scaffolds Production Using 3D Plotting

The prepared PCL/HA composite solutions with various HA contents (0 wt %, 10 wt %, 15 wt %, and 20 wt %) were extruded through a nozzle with a diameter of ~640 µm and then deposited at a constant speed of 3 mm/s in an ethanol (EtOH) bath at room temperature using a computer-controlled robot (Ez-ROBO5, Iwashita, Japan). To produce 3-dimensionally interconnected macropores in a periodic pattern, the extruded PCL/HA filaments were deposited at a stacking sequence of 0°/90°, while the distance between the deposited PCL/HA filaments was 500 µm for all the samples.

### 3.3. Macro/Micro-Porous Structure Evaluation

The macro/micro-porous structures of the PCL/HA composite scaffolds, which had dimensions of ~14 mm × 14 mm × 1.4 mm, produced with various HA contents (0 wt %, 10 wt %, 15 wt %, and 20 wt %), were characterized by field emission scanning electron microscopy (FE-SEM; JSM-6701F; JEOL Techniques, Tokyo, Japan). The dimensions of the 3-D macropores, PCL/HA filaments, and micropores formed in the PCL/HA filaments were roughly estimated from FE-SEM images of the samples. Twenty regions were measured to obtain the mean and standard deviation.

The overall porosity (*p*) of the macro/micro-porous scaffolds was calculated by considering their apparent density (ρ_a_) and strut density (ρ_s_) as follows:

*p* = 1 − (ρ_a_/ρ_s_) (1)

The apparent density (ρ_a_) of the porous scaffolds was computed by measuring their mass (m) and volume (V) (i.e., ρ_a_ = m/V). The strut density (ρ_s_) was also computed by considering the theoretical density of the PCL (1.145 g/cm^3^) and HA (3.14 g/cm^3^) [34], as follows:

*p* = X_PCL_ρ_PCL_ + X_HA_ρ_HA_ (2)

where X_PCL_ and X_HA_ indicate the volume fractions of the PCL and HA, respectively. Five specimens were tested to obtain the mean and standard deviation.

### 3.4. TGA Analysis

The final content of the HA particles in the porous PCL/HA scaffolds produced with various HA contents (10 wt %, 15 wt %, and 20 wt %) was determined by thermogravimetric analysis (TGA; TA Instruments, New Castle, DE, USA). The scaffolds were heated up to 500 °C at a heating rate of 10 °C/min in a flowing nitrogen atmosphere. The weight losses of the scaffolds during the tests were monitored and used to calculate the final HA contents. One sample was tested for each composition.

### 3.5. Mechanical Properties Evaluation

The mechanical properties of the macro/micro-porous PCL/HA composite scaffolds produced with various HA contents (0 wt %, 10 wt %, 15 wt %, and 20 wt %) were tensile strength tests. Specimens with dimensions of ~7.7 mm × 4.5 mm × 1.4 mm were uniaxially elongated at a cross-head speed of 1 mm/min using a screw-driven load frame (Oriental Testing Machine Co, Siheung-si, Korea). In addition, compressive strength tests were conducted using the samples with dimensions of ~4.4 mm × 1.4 mm × 5.2 mm. The stress versus strain responses of the specimens during the tests were recorded. The ultimate tensile strength and elastic modulus were calculated from the stress-strain curves. Five specimens were tested to obtain the mean and standard deviation.

### 3.6. In Vitro Cytocompatibility Evaluation

The in vitro cytocompatibility of the macro/micro-porous PCL/HA scaffolds produced with various HA contents (0 wt %, 10 wt %, 15 wt %, and 20 wt %) was evaluated using a pre-osteoblast cell line (MC3T3-E1; ATCC, CRL-2593, Rockville, MD, USA) [26]. Prior to the cell seeding, the samples were sterilized with 70% ethanol for 30 minutes and dried on a clean bench under ultraviolet (UV) irradiation for 12 h. The preincubated cells were plated at a density of 5 × 10^4^, 2 × 10^4^, and 1 × 10^4^ cells/mL for the initial cell attachment, proliferation and differentiation tests, respectively [26,35]. A 12-well culture plate (SPL Life Sciences Co., Ltd., Gyeonggi-do, Korea) with walls having a diameter of 20 mm and a depth of 18 mm was used for this evaluation. The MC3T3-E1 cells were cultured in a humidified incubator in an atmosphere containing 5% CO_2_ at 37 °C. A minimum essential medium (α-MEM: Welgene Co., Ltd., Seoul, Korea) supplemented with 10% fetal bovine serum (FBS), 1% penicillin-streptomycin, 10 mM β-glycerophosphate (Sigma, Taufkirchen, Germany), and 10 µg mL^−1^ ascorbic acid was used as the culturing medium. In addition, a basal medium (α-MEM: Welgene Co., Ltd., Seoul, Korea) was also used for comparison purposes.

The morphologies of the attached cells on the macro/micro-porous PCL/HA scaffolds produced with various HA contents (0, 5, 10, and 20 wt %) after 24 h of culture were examined by confocal laser scanning microscopy (CLSM; C1 PLUS, Nikon, Tokyo, Japan). For these CLSM observations, the cultured cells were dyed with Alexa Fluor 546 phalloidin (Thermo Fisher Scientific Inc., Eugene, OR, USA) and ProLong Gold antifade reagent with DAPI (Thermo Fisher Scientific Inc., Eugene, OR, USA). The stained substrates were placed on a cover slide without further treatments, and the cell morphology was observed. Two samples were examined for each composition.

The cell proliferation rate was examined using a MTS (methoxyphenyl tetrazolium salt) assay with 3-(4,5-dimethylthiazol-2-yl)-5-(3-carboxymethoxyphenyl)-2-(4-sulfophenyl)-2H-tetrazolium (MTS, Promega, Madison, USA) for mitochondrial reduction. The quantity of the formazan product, which is measured by the absorbance at 490 nm using a micro-reader (Model 550; Biorad, Hercules, CA, USA), is directly proportional to the number of living cells in the culture. Five samples were tested for each test.

Cell differentiation was assessed using an alkaline phosphatase (ALP) activity test, in which 10 mM β-glycerophosphate (β-GP) and 50 µg/mL ascorbic acid (AA) were added to the culture medium. After culturing for 7 days, p-nitrophenol (pNP) production was colorimetrically measured at an absorbance of 405 nm using a micro reader (Model 550; Biorad, Hercules, CA, USA). During this reaction, pNPP was converted to pNP in the presence of ALP; therefore, the pNP production rate was proportional to the ALP activity. Five samples were tested for each test.

### 3.7. In Vitro Apatite-Forming Ability Evaluation

The in vitro apatite-forming bioactivity of the macro/micro-porous PCL/HA scaffolds produced with various HA contents (0 wt %, 10 wt %, 15 wt %, and 20 wt %) was characterized using simulated body fluid (SBF) solution [32,33]. The porous scaffolds were immersed in the SBF solutions and then placed inside an incubator at a controlled temperature of 37 °C for 3 days and 7 days. Two samples were tested for each composition. The formation of apatite layers on the porous scaffolds was examined by FE-SEM and energy dispersive spectroscopy (EDS) attached to the FE-SEM.

### 3.8. Statistical Analysis

All experimental results were expressed as the mean ± standard deviation (SD). Five samples were tested for each test. The difference between the two groups was determined using a one-way analysis of variance (ANOVA) and *p* < 0.05 and *p* < 0.01 were considered to be statistically significant (* *p* < 0.05 and ** *p* < 0.01).

## 4. Conclusions

Porous PCL/HA composite scaffolds with a controlled macro/micro-porous structure were successfully produced using NIPS-based 3D plotting, in which PCL/HA solutions could be effectively solidified as microporous PC/HA filaments by means of the exchange of solvent (THF) and non-solvent (EtOH). Regardless of the HA contents, all of the PCL/HA composite scaffolds had 3-dimensionally interconnected macrochannels surrounded by highly microporous PCL/HA struts that were constructed in a controlled periodic pattern. In addition, with an increase in HA content, the mechanical properties (i.e., ultimate tensile strength and compressive yield strength) and apatite-forming ability increased significantly. These findings suggest that the NIPS-based 3D plotting technique is very useful in the production of porous PCL-based composite scaffolds with the controlled macro/micro-porous structure (e.g., high porosity, 3-dimensionally interconnected macrochannels and micropores), high mechanical properties, and good bioactivity. In addition, the present technique can be applied to a variety of biocompatible, biodegradable polymers, even with bioactive inorganic phases, thus finding very useful applications in bone tissue regeneration.

## Figures and Tables

**Table 1:** The porous structure (overall porosity, diameter of strut, widths of macropore, and size of micropore) of the macro/micro-porous PCL/HA composite scaffolds produced with various HA contents (0 wt %, 10 wt %, 15 wt %, and 20 wt %).

HA Content [wt %]	0	10	15	20
Overall Porosity [vol %]	78.4 ± 1.2	77.0 ± 3.5	77.9 ± 1.0	78.3 ± 1.6
Diameter of Strut [µm]	219 ± 16	270 ± 3	271 ± 21	273 ± 17
Widths of Macropore [µm]	248 ± 16 × 83 ± 18	184 ± 5 × 75 ± 12	183 ± 7 × 67 ± 9	184 ± 4 × 174 ± 6
Size of Micropore [µm]	2.8 ± 1.2	2.9 ± 1.1	2.3 ± 1.0	2.1 ± 0.8

**Table 2:** Alkaline phosphatase (ALP) activity of the MC3T3-E1 cells that were cultured for 7 days on the macro/micro-porous PCL/HA composite scaffolds (HA content = 0 wt %, 10 wt %, 15 wt %, and 20 wt %) using a basal medium.

HA Content [wt %]	0	10	15	20
ALP Acivity [p-NP/mg protein/h]	0.0245 ± 0.0025	0.0288 ± 0.002	0.0342 ± 0.003	0.0284 ± 0.005
